# 

**Published:** 1878-06

**Authors:** 


### Table of Contents.

### ORIGINAL COMMUNICATIONS.

Puerperal Eclampsia. *By W. B. Prather, M.D., Florence, Ga*... 129

### REPORTS OF SOCIETIES.

Atlanta Academy of Medicine........................................ 130

### SELECTIONS.

Rotheln Rubeola—German Measles........................................ 135
Recent Progress in Physiology........................................ 139
Diaphragmatic Hernia........................................ 145
Substitute for the Mother's Milk During Infancy........................................ 146
Mistletoe as an Oxytoxic........................................ 150
A Case of Vicarious Menstruation........................................ 152
Rapid Dilatation of the Female Urethra for the Relief of Vesical Irritability........................................ 153
The Vomiting of Pregnancy and its Treatment........................................ 156

### EDITORIAL.

Criminal Abortion........................................ 165
European Correspondence........................................ 169
Editorial Correspondence........................................ 180, 183

### BIBLIOGRAPHICAL.

Prescription Writing........................................ 185
Amputation of Cervix Uteri........................................ 185
Objections to use of Carbolic Acid in Treatment of Piles........................................ 185
Pamphlets Received........................................ 186

### EXCERPTA.

Treatment of Epilepsy........................................ 186
Items........................................ 187
The External use of Tincture of Belladonna in Night Sweating........................................ 187
Carron Oil in Anal Fissure........................................ 187
Metric System in the United States Army........................................ 188
Nitric Acid for Whooping Cough........................................ 188
Poison in the Nursery........................................ 188
Treatment of Diphtheria........................................ 189
Professional Secrets........................................ 189
Public Analysts........................................ 190
Death of Prof. Francis G. Smith........................................ 190
Invisible Ink for Postals........................................ 190
The Coming Duties of the Accoucher........................................ 190
To Keep the Cord from Slipping in Extracting the Placenta........................................ 191
A Good Cleansing Fluid........................................ 192
Local Treatment of Acne........................................ 192

